# Experiences of stigmatization and its impacts among individuals living with hereditary diseases and family members in Portugal: an exploratory study

**DOI:** 10.1007/s12687-025-00782-7

**Published:** 2025-02-28

**Authors:** Joana Valentim, Milena Paneque, Álvaro Mendes

**Affiliations:** 1 Faculty of Psychology and Educational Sciences of University of Coimbra, Coimbra, Portugal; 2 CGPP – Centre for Predictive and Preventive Genetics, IBMC – Institute for Molecular and Cell Biology, University of Porto, Porto, Portugal; 3 i3S – Institute for Research and Innovation in Health, University of Porto, Porto, Portugal; 4 ICBAS – School of Medicine and Biomedical Sciences, University of Porto, Porto, Portugal

**Keywords:** Hereditary diseases, Stigma, Health psychology, Discrimination, Family members

## Abstract

Stigma is defined as the perception of an undesirable attribute that leads to discrimination against individuals and groups. Stigmatisation is often triggered due to visible physical or cognitive differences. Although the literature consistently highlights the (fear of) stigmatisation as a significant concern among individuals living with hereditary conditions, no studies in Portugal have specifically provided evidence on this issue. This study aims to address this gap by examining the experiences and impact of stigma on individuals and families affected by hereditary diseases in Portugal. After receiving ethics approval, a total of 216 participants, including affected individuals, asymptomatic carriers and family members from families with a range of hereditary conditions, were recruited through patient support associations. Participants completed an online questionnaire via Limesurvey. Data were analysed through Exploratory Factor Analysis (EFA), median comparison tests, and thematic analysis. Of the participants, 78.7% were women, 55.6% had a university degree, and 20.4% were aged between 42 and 47 years. Findings indicate that stigma impacts individuals across various domains, including social interactions, institutional settings, the workplace, and healthcare. EFA identified a bi-factorial model of stigma, comprising Stigma Experiences and Perceived Support subscales, and the overall scale demonstrated high internal consistency (α = .879). Women and younger participants reported higher levels of stigma. Religiosity and humor emerged as key coping strategies. This study is the first in Portugal to assess stigma among individuals living with hereditary conditions. Our findings contributed to validating a measurement instrument, identified sociodemographic variations, and examined the psychosocial dimensions of stigma among affected patients. These findings highlight the need for comprehensive strategies to address and mitigate stigma, improve support systems, and enhance the well-being and healthcare experiences of individuals and families impacted by hereditary diseases.

**Supplementary Information:**

The online version contains supplementary material available at 10.1007/s12687-025-00782-7.

## Introduction

Stigma refers to discrimination and devaluation of individuals based on perceived undesirable traits (Goffman 1963). In the context of health, stigma arises from adverse social judgment associated with a health condition, leading to exclusion, blame, or rejection. This judgment is based on enduring characteristics of identity associated with a health problem or condition, often lacking adequate medical justification (Weiss et al. 2006). According to Link and Phelan (2001), stigma involves labelling, stereotyping, separating "us" from "them," loss of status, and discrimination, with the presence of social power enabling these processes. Stigma manifests as enacted stigma (social rejection and discrimination) and felt stigma (shame and fear of future discrimination) (Scambler 1998).

Individuals with hereditary diseases often experience stigma related to visible physical or cognitive alterations, which can significantly impact their quality of life and ability to manage their condition (Liu et al. 2017; Subu et al. 2021). This can occur across various contexts, including romantic relationships, family interactions, hospital settings, and the workplace (Bombard et al. 2007; Sexton et al. 2019; Faure et al. 2019). Felt stigma—often more distressing than enacted stigma—can lead to reduced self-confidence, embarrassment, and fear of future discrimination (Bombard et al. 2007; Erwin et al. 2010). Symptoms' visibility also plays a crucial role in experiences of stigma, as visible signs of illness may trigger embarrassment and social rejection (Joachim and Acorn 2003; Sexton et al. 2019). The psychological toll of stigma in this population is well-documented. Individuals with higher levels of stigma report greater distress, while those with stronger self-esteem and a sense of control experience less anxiety (Vodermaier et al. 2010). Predictive genetic testing has made early diagnosis of hereditary diseases possible in certain cases. While a positive genetic result can lead to better disease management and emotional relief, it may also result in career restrictions, higher insurance premiums, and social discrimination (Etchegary 2007; Bonner et al. 2018; Granero-Molina et al. 2020). Patients often struggle with identity changes and self-perception after receiving a genetic diagnosis (Geerts et al. 2017). However, familiarity with the disease within the family may reduce anxiety in the context of genetic testing (Paneque et al. 2009). Despite the challenges, some patients and families find relief in the certainty provided by genetic testing (Pereira et al. 2024). Knowing the genetic cause can reduce personal guilt and help manage stigma by offering an explanation for the condition (Sankar et al. 2006; Manz 2016).

To cope with experiences of stigma, people adopt various approaches, such as reducing their engagement in stigmatizing environments, and confronting or delegitimizing the source of stigma (West 1984; Sankar et al. 2006). Comparison with others is another common coping strategy to mitigate feelings of stigma (Sexton et al. 2019). In a Portuguese study, young adults seeking pre-symptomatic testing for hereditary Transthyretin Amyloidosis navigated the stigma associated with their disease by seeking support from significant others and limiting discussions about the disease to a select group to mitigate potential prejudice (Pereira et al. 2024). In some cases, patients report receiving sympathy rather than stigma from their social circles (Etchegary 2007). Despite the prevalence of stigma in hereditary diseases, no specific psychometric tool exists to assess these experiences in this population.

The influence of sociodemographic factors on the enactment of stigma is inconsistent. While some research has found no significant association between stigma and variables such as gender or ethnicity (Boileau et al. 2020), other studies suggest that women and younger individuals experience higher levels of stigma compared to men and older adults (Vodermaier et al. 2010; Ayode et al. 2016). Formal educational level also seems to influence perceptions of stigma, with individuals with higher educational levels reporting higher perceptions of stigmatization (Liu et al. 2017). In a study conducted by Boileau et al. (2020), internal factors such as disease stage and health-related quality of life were found to explain approximately 20.2% of the variance in stigma within their proposed model.

Despite numerous studies exploring stigma experiences related to health, particularly in the context of mental illnesses (e.g. Whitley and Campbell 2014) and sexual transmissive diseases (e.g. Molina and Ramirez-Valles 2013; Lodi et al. 2023; Nilsson Schönnesson et al. 2024), the experiences of stigma among individuals with hereditary diseases are still scarcely reported, particularly within the Portuguese context. This study aims to explore stigma experiences among individuals with hereditary diseases and family members in Portugal. Specifically, it seeks to: (1) characterize stigma experiences and discrimination considering participants' sociodemographic characteristics and disease features (e.g., age at diagnosis, age of symptom onset); (2) identify the contexts in which stigma occurs and the actors involved; (3) determine the impacts of stigma on the patient and/or family psychologically (e.g., sadness/depression, parental guilt, fear, anger/distress, shame, among others), socially (e.g. exclusion by others), and in terms of quality of life; (4) understand how individuals with hereditary diseases or family members cope with stigma and what resources they turn to in order to deal with it, and (5) conduct an Exploratory Factor Analysis (EFA) to identify the latent variables underlying the stigma experience in this population and contribute to the development of a psychometric instrument for assessing stigma experiences in this specific group.

## Methods

This study uses a descriptive, exploratory, and non-experimental approach. It has received ethical approval from the CECRI of i3S (22/2023).

### Recruitment

Participants included individuals with a clinical diagnosis of hereditary diseases, family members of those with a clinical diagnosis or asymptomatic carriers, all residing in Portugal. Participants were recruited through associations supporting patients and families with hereditary diseases. We contacted 39 associations asking them to advertise the study to their associates. Nineteen agreed and advertised the study through social media, mailing lists, or in-person meetings. Our aim was to recruit as many participants as possible from diverse health backgrounds. Although the symptoms and characteristics of the various diseases differ, participants are united by the hereditary nature of their own diseases or the diseases affecting their family members.

### Instrument

An online questionnaire, tailored for individuals diagnosed with hereditary diseases, family members, and asymptomatic carriers, was developed based on literature on stigma and its psychosocial impacts. Participants were allowed to complete multiple versions of the questionnaire as applicable to their situation. Content validity was reviewed by two clinical geneticists, a genetic counselor, a psychologist, and a representative from a patient support association. Revisions addressed sociodemographic details, informed consent options, and language clarity. The questionnaire consisted of three sections: (1) sociodemographic data; (2) targeted questions according to participant status assessing disease-related experiences in various contexts. Topics of questions included: family and interpersonal relationships, social and friendship dynamics, work and professional challenges, societal perceptions and support, psychological and emotional well-being, coping strategies and adaptation, and disclosure and privacy concerns; (3) an open-ended section for participants to provide additional relevant information. For access to the complete questionnaire, please refer to the Suplementary Information. To ensure data anonymity, the Ethics Committee recommended reporting participants' age in ranges instead of specific years.

The final questionnaire comprised 33 items. A 5-point Likert scale was used, with higher scores indicating greater stigmatization. Face validity was evaluated through a pilot study, leading to clarification of specific terms and items. The questionnaire was tested with four participants (two women, mean age 58), three of whom had hereditary diseases, and one was a family member of a patient. Participants were recruited through the authors’ networks and the Portuguese Association of Amyotrophic Lateral Sclerosis and the Portuguese Association of Huntington’s Disease.

### Data collection

Data was collected via the platform Limesurvey between February and April 2024. Participants accessed the survey through a link or QR code and provided informed consent before beginning. The survey took approximately 10 min to complete and no compensation was provided.

### Data analysis

**Fig. 1:**
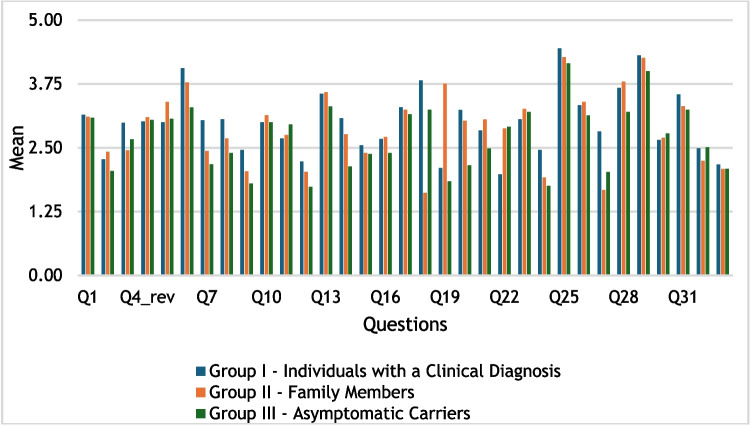
Comparison of mean score responses across the three questionnaires

The sample was divided into three groups: Group I—individuals with a clinical diagnosis (n = 116), Group II—family members (n = 74), and Group III—asymptomatic carriers (n = 45). The mean responses for each questionnaire were calculated and ordered from highest to lowest. A comparison of the mean scores between the three questionnaires was then developed and visualized in a graph (Fig. 1). An EFA was conducted to identify latent variables underlying stigma experiences, using only the patients' version of the questionnaire due to small sample sizes in other groups. JASP software version 0.18.3 and SPSS versions 27 and 29 were used for statistical analysis. The suitability of the data for factor analysis was verified using the Kaiser–Meyer–Olkin (KMO) measure and Bartlett’s test of sphericity (Field 2009). To examine the latent variables underlying stigma experiences across sociodemographic characteristics, we performed median comparisons using the Mann–Whitney test due to small sample sizes and non-parametric data and Pearson and Spearman correlations. The participants' responses to the open-ended questions of the questionnaire were analyzed thematically (O’Cathain and Thomas 2004). We used an inductive approach with reference to a broad psychosocial framework, aiming to identify recurring themes that reflected participants' individual and interpersonal experiences with stigmatization. The first and last authors separately read all the open-ended responses and manually mapped the key topics describing the thematic content of the comments. Both met to discuss their proposals and, when doubts arose, the second author was included until an agreement was reached.

## Results

### Demographics

Of the 299 participants who accessed the study, 216 completed the full questionnaire, resulting in a completion rate of 72.2%. The majority of the participants were women (78.7%), were married or in a relationship (61.1%), held a university degree (55.6%), lived in urban areas (77.8%), were employed (69.9%), and aged between 42 and 47 years old (20.4%). 75.5% of participants had children, while 27.8% expressed a desire to have children (Table 1).

**Table 1:** Sociodemographic characteristics of participants

Sociodemographic characteristics	Total (*n* = 216)		Group I (*n* = 116)		Group II (*n* = 74)		Group III (*n* = 45)	
	*n*	%	*n*	%	*n*	%	*n*	%
Gender								
Woman	170	78.7	90	70.6	55	74.3	41	91.1
Man	46	21.3	26	22.4	19	25.7	4	8.9
Age category								
18 – 23 years	5	2.3	3	2.6	0	0	2	4.4
24 – 29 years	11	5.1	6	5.2	1	1.4	5	11.1
30 – 35 years	14	6.5	5	4.3	4	5.4	7	15.6
36 – 41 years	38	17.6	21	18.1	13	17.6	6	13.3
42 – 47 years	44	20.4	25	21.6	13	17.6	9	20
48 – 53 years	42	19.4	19	16.4	18	24.3	10	22.2
54 – 59 years	34	15.7	20	17.2	15	20.3	3	6.7
60 – 65 years	15	6.9	11	9.5	4	5.4	1	2.2
+ 66 years	13	6	6	5.2	6	8.2	2	4.4
Marital status								
Single	43	19.9	24	20.7	8	10.8	15	33.3
Married/partnered	132	61.1	67	57.8	50	67.6	25	55.6
Divorced	32	14.8	21	18.1	12	16.2	4	8.9
Widowed	9	4.2	4	3.4	4	5.4	1	2.2
Children^a^	163	75.5	82	70.7	68	91.9	26	57.8
How many children ^b^								
1	60	27.8	29	25	23	31.1	10	22.2
2	81	37.5	40	34.5	35	47.3	14	31.1
3	15	6.9	8	6.9	8	10.8	2	4.4
4	5	2.3	4	3.4	1	1.4	0	0
5	2	0.9	1	0.9	1	1.4	0	0
Desire to have children^a^	60	27.8	27	23.3	22	29.7	14	31.1
Highest educational level								
Middle school	26	12	18	15.5	9	12.2	0	0
High School	70	32.4	47	40.5	18	24.3	10	22.2
University or postgraduate degree	120	55.6	51	44	47	63.5	35	77.8
Employment								
Unemployed	19	8.8	16	13.8	1	1.4	3	6.7
Student	5	2.3	3	2.6	0	0	2	4.4
Employed	151	69.9	67	57.8	61	82.4	36	80
Informal caretaker	5	2.3	3	2.6	2	2.7	1	2.2
Retired	34	15.7	25	21.6	0	0	3	6.7
Medical leave	2	0.9	2	1.7	0	0	0	0
Household								
0	14	6.5	8	6.9	3	4.1	3	6.7
1	71	32.9	45	38.8	18	24.3	17	37.8
2	60	27.8	31	26.7	22	29.7	8	17.8
3	60	27.8	27	23.3	27	36.5	15	33.3
+ 3	11	5.1	5	4.3	4	5.4	2	4.4
Area of residence								
Rural	48	22.2	34	29.3	9	12.2	7	15.6
Urban	168	77.8	82	70.7	65	87.8	38	84.4

^a^Reflects the number and percentage of participants answering “yes” to this question

^b^Of those who responded affirmatively to having children

The study identified 51 distinct hereditary conditions, revealing a heterogeneous distribution among the participants. The five most common conditions reported were hereditary psoriasis (14.4%), Huntington’s disease (13%), Machado-Joseph’s disease (7.9%), Hemochromatosis (7.9%), and Neurofibromatosis type 1 (7.4%). Four participants had not yet received a concrete diagnosis, and 25 of the reported conditions were unique to a single respondent (Table 2).

**Table 2:** Table of hereditary conditions present

Hereditary condition	Frequency	Percentage
1. Hereditary Psoriasis	31	14.4
2. Huntington's Disease	28	13.0
3. Machado-Joseph Disease	17	7.9
4. Hemochromatosis	17	7.9
5. Neurofibromatosis type 1	16	7.4
6. Tuberous Sclerosis	14	6.5
7. Achondroplasia	10	4.6
8. Parkinson's Disease	8	3.7
9. BRCA2	7	3.2
10. Ataxia	4	1.9
11. Common Variable Immunodeficiency	4	1.9
12. Still without a concrete diagnosis	4	1.9
13. Amyotrophic Lateral Sclerosis	3	1.4
14. BRCA1	3	1.4
15. Friedreich's Ataxia	3	1.4
16. Congenital Central Hypoventilation Syndrome	3	1.4
17. Ankylosing Spondylitis	2	0.9
18. Hereditary Cancer	2	0.9
19. Lynch Syndrome	2	0.9
20. ACAN Gene Mutation	2	0.9
21. Cartilage-Hair Hypoplasia	2	0.9
22. Chronic Granulomatous Disease	2	0.9
23. Spondylometaphyseal Dysplasia	2	0.9
24. Multiple Epiphyseal Dysplasia	2	0.9
25. Bone Dysplasia	2	0.9
26. Psoriatic Arthritis	1	0.5
27. Severe Combined Immunodeficiency Syndrome	1	0.5
28. Prader-Willi Syndrome	1	0.5
29. Ondine's Syndrome	1	0.5
30. Gardner Syndrome	1	0.5
30. Brugada Syndrome	1	0.5
32. Familial Amyloid Polyneuropathy	1	0.5
33. Pycnodysostosis	1	0.5
34. Multiple Osteochondromatosis	1	0.5
35. Ovarian Cancer (RAD51C genetic mutation)	1	0.5
36. Systemic Lupus Erythematosus	1	0.5
37. Li-Fraumeni Syndrome	1	0.5
38. X-Linked Hypophosphatemic Rickets (XLH)	1	0.5
39. Hypophosphatasia	1	0.5
40. Hypochondroplasia	1	0.5
41. Polymyositis	1	0.5
42. Bone Dysplasia Type II—Desbuquois Syndrome	1	0.5
43. Diastrophic Dysplasia	1	0.5
44. Cleidocranial Dysplasia	1	0.5
45. Leri-Weill Dyschondrosteosis	1	0.5
46. Congenital Adenosine Deaminase 2 (ADA2) Deficiency	1	0.5
47. Hypertrophic Cardiomyopathy (HCM)	1	0.5
48. Charlevoix-Saguenay Ataxia (ACS)	1	0.5
49. Ataxia AOA4	1	0.5
50. Fanconi Anemia	1	0.5
51. X-Linked Agammaglobulinemia	1	0.5
Total	216	100.0

Regarding participants' engagement with patient associations, 28.7% had monthly contact with an association, 13.9% had contact more than once a week, 7.4% had weekly contact, 19% were members with no contact, 21.3% were not associated, and 9.7% were unaware of support institutions for their condition.

Of the 216 participants, 116 were diagnosed with a hereditary disease. In this group, 70.6% were woman, 57.8% were married or in a relationship, and 57.8% were employed. No significant gender differences were observed across socio-demographic variables such as education level, professional status, or level of involvement with patient associations.

### Preliminary data distribution analysis

The preliminary analysis examined the distribution of mean scores across the questionnaire for the three groups: Group I (individuals with a clinical diagnosis), Group II (family members), and Group III (asymptomatic carriers). Figure 1 presents a comparison of mean responses for each question across these groups.

A higher mean score indicates stronger agreement with the statements. Visual inspection of the graph shows that six out of the seven highest-scoring questions were consistent across all groups. Questions 25 and 29 received the highest ratings, indicating that participants across all groups felt comfortable discussing the disease with healthcare professionals (M ≥ 4.16) and actively sought information to manage the disease (M ≥ 4). Other questions with high agreement involved open communication within the family (Q6), support from friends (Q13), comfort with others knowing about the disease (Q18), and improved coping over time (Q28). In contrast, the lowest-scoring questions, with mean scores of M ≤ 2.46, reflect lower levels of agreement with the statements. These included facing prejudice at work due to the disease (Q9), believing that knowing about the disease would affect forming friendships (Q12), avoiding new friendships to avoid explaining the disease (Q19), feeling that others avoid them because of the disease (Q24), and perceiving adequate support from Social Security services (Q33).

Overall, psychosocial experiences appeared to be similar across all groups. Questions with high mean scores across all groups reflected agreement with statements about positive psychosocial experiences of living with the disease. Conversely, questions with low mean scores suggested disagreement with statements related to stigma and other negative psychosocial aspects. The only clear negative experience was the perceived lack of support from Social Security.

### EFA for group I (individuals with a clinical diagnosis)

An EFA was performed on data from the group of diagnosed individuals to explore the latent variables associated with stigma. The KMO measure was 0.748, indicating adequate sampling, and Bartlett’s test of sphericity was significant (χ^2^(528) = 5,137.105, *p* < .001) suggesting that the correlations between questions were sufficiently large for EFA. We conducted parallel analysis based on factor analysis to ascertain the number of factors and subsequently applied oblique (Promax) rotation to obtain a more interpretable factor structure, which initially identified nine factors. After refining the model by excluding low-loading questions and cross-loadings, a two-factor solution emerged, explaining 47.7% of the total variance. Factor 1 (32.2% of the variance) pertained to stigma experiences, while Factor 2 (15.5%) related to dissatisfaction with perceived social and institutional support (Table 3). Due to positively phrased items, the scoring for Factor 2 was reversed to align with the overall stigma measure, so the total scale score reflected higher stigma experiences and lower perceived support.

**Table 3:** Results from an exploratory factor analysis of the stigma experiences and perceived support scale (SEPSS)

SEPSS item	Factor loading	
	1	2
Factor 1: Stigma Experiences		
24. Some people avoid me because of my disease	**0.82**	0.15
3. I feel that knowing about my disease would affect someone's desire to have an intimate relationship with me	**0.79**	0.12
27. Sometimes, I feel ashamed because of my disease	**0.76**	− 0.03
20. I have experienced negative reactions from other people in social situations because of my disease	**0.75**	0.00
12. I feel that knowing about my disease would affect someone's desire to want to be my friend	**0.71**	0.09
9. I face prejudice or discrimination in my workplace due to my disease	**0.65**	− 0.12
17. I often feel sad because of my disease, even when I try to be optimistic	**0.65**	− 0.09
32. I have felt the need to hide information about the disease	**0.62**	− 0.04
14. My disease has negatively affected/affects my participation in social activities	**0.55**	− 0.12
Factor 2: Perceived Support		
1. I feel satisfied with my social support network. (R)	0.24	**0.90**
15. Society provides me with the necessary support to deal with challenges related to my hereditary disease. (R)	− 0.09	**0.63**
33. I feel that Social Security provides me with the necessary support to deal with my disease. (R)	0.07	**0.59**
10. I feel that at my workplace, I have the necessary support to deal with challenges related to my disease. (R)	− 0.09	**0.56**
26. Healthcare professionals understand the challenges I face in society due to my hereditary disease. (R)	− 0.10	**0.52**

*N* = 116. The extraction method was parallel analysis based on factor analysis with an oblique (Promax) rotation. Factor loadings above 0.40 are in bold. Reverse-scored items are denoted with an (R)

Cronbach's alpha coefficients were calculated to determine the internal consistency of the scale. The overall scale demonstrated good internal consistency, with an alpha coefficient of α = 0.879. Factor-specific analyses revealed that Factor 1 (stigma experiences) had a strong internal consistency (α = 0.897), while Factor 2 (perceived support) exhibited acceptable reliability (α = 0.775).

### Stigma experiences, perceived support, and sociodemographic variables

Participants from Group I (individuals with a clinical diagnosis) had a mean overall score of 40.85 ± 11.98 on the scale, with a mean of 25.06 ± 8.79 for stigma experiences and 15.79 ± 4.27 for perceived support.

No significant differences were found on the overall scale based on sociodemographic factors such as place of residence, employment, or marital status (all *p* > .061). Although women had a higher median score on the overall scale (*Mdn* = 42.5) compared to men (*Mdn* = 37.5), this difference was not statistically significant (*p* = .096). However, in the stigma experiences subscale, women reported significantly more stigma than men (*Mdn* = 27 vs. *Mdn* = 22, *p* = .011). No gender differences were observed in the perceived support subscale. Additionally, a weak negative correlation was found between the total scale score and both the age of diagnosis (*r* = −0.30, *p* = .002) and age of symptom onset (*r* = −0.27, *p* = .007). These associations were particularly evident in the stigma experiences subscale (age of diagnosis (*r* = −0.35, *p* < .001) and age of symptom onset (*r* = −0.31, *p* = .002)) but were absent in the perceived support subscale (age of diagnosis (*r* = −0.08, *p* = .402) and age of symptom onset (*r* = −0.10, *p* = .330)), suggesting that younger age at diagnosis or symptom onset is associated with higher experiences of stigma.

### Qualitative analysis of the open field

Thirty-seven participants (17.3%) provided written responses, which were thematically analyzed. Five themes emerged: (1) challenges related to heredity and diagnosis, (2) emotional, familial, and social impacts, (3) lack of social and institutional support, (4) stigma experiences, and (5) coping strategies. Participants described significant emotional struggles, concerns about sharing genetic information with their children, and frustration with insufficient support from Social Security and healthcare providers. Stigma experiences were reported in various contexts, including workplaces and healthcare settings, and participants coped with their challenges through faith and humor.

“*Rare diseases in Portugal are stigmatized and hidden. A person diagnosed with a rare disease in the workplace does not see their working hours reduced, nor the possibility, when necessary, of being on medical leave without salary cuts, despite facing significant economic difficulties*” (Woman, aged between 48 and 53 years, diagnosed with Common Variable Immunodeficiency).

“*The disability verification service of social security in [place of residence] treated me as if I were begging (stigma) from the state. In both verifications/evaluations, the doctors observed me (looking at the computer) for ten minutes, I felt humiliated (treated as if I were a marginal), and the reports from the team of doctors were ignored*” (Male, aged between 60 and 65 years, diagnosed with Huntington’s disease).

"*I usually say that now, my life runs on wheels, referring to the wheelchair, sarcastically*" (Woman, aged between 54 and 59 years, diagnosed with Machado Joseph's Disease).

## Discussion

This study aimed to examine the experiences, attitudes, and impact of stigma on individuals diagnosed with hereditary diseases and family members in Portugal. This study adds to the growing body of literature on the psychosocial challenges and stigma-related experiences that not only affect individuals diagnosed with hereditary diseases but also asymptomatic carriers and family members. Our preliminary analysis suggests a consistent response pattern across all groups: individuals with a clinical diagnosis, family members, and asymptomatic carriers. It appears that all groups exhibit similar levels of agreement and disagreement with questions pertaining to the same psychosocial and stigmatizing experiences. This information aligns with the literature, which shows that stigmatization affects not only individuals diagnosed with a condition (Munro et al. 2022) but also extends to those who are positive carriers (Baynam et al. 2024) and family members (Magliano et al. 2014).

The results indicate that, in general, the questions reflecting positive experiences with living with hereditary diseases had higher mean scores (agreement with the statement) across all groups, while questions depicting stigma and difficulties with the disease had lower mean scores (disagreement with the statement). This pattern suggests that participants generally disagreed with statements implying that the disease negatively influenced their interactions with others, including friends, family, and colleagues. One possible explanation for this outcome may be the fact that our participants were recruited through patient support associations, which offer social, educational, and informational resources to individuals and their families (Patterson et al. 2023). These individuals may represent a group that benefits from the protective role of these support organizations. Studies show that access to support networks, even indirectly, can reduce perceived public stigma (Zhu et al. 2017). As our study was advertised through patient associations, even those participants who are not directly involved with these organizations may still benefit from the information and resources they disseminate. Thus, engagement with these associations may foster a perception of support and potentially diminish stigma experiences.

However, there is a shared negative psychosocial experience across all groups regarding the perception of support provided by Social Security: all groups perceive this support as insufficient. Social Security is an essential institution, with one of its objectives being to support individuals with rare diseases through various forms of assistance, such as financial aid, and social responses tailored to the needs of this population (Diário da República 2015). According to Llubes-Arrià et al. (2022), some barriers in the diagnostic process for people with rare diseases and their families include inadequate and insufficient resources, financial support, and other support systems. Due to the perceived insufficient support from Social Security, individuals with rare diseases may encounter heightened challenges in managing their conditions, potentially leading to a diminished quality of life. The lack of adequate support may exacerbate the psychosocial burden on these individuals, hindering their ability to effectively cope with the disease and negatively impacting their overall well-being.

The EFA’s results identify stigma experiences and perceived support as latent variables, unveiling distinct dimensions underlying the nature of stigma experiences within our study population. The instrument demonstrates good psychometric properties such as construct validity and scale reliability. These results contribute to the initial validation of the psychometric instrument and suggest it is a robust tool for assessing stigma experiences among individuals with hereditary diseases in the Portuguese context. Moreover, the negative correlation between stigma experiences and perceived support is consistent with findings from other studies, which indicate that greater support correlates with lower levels of perceived stigma (Lei et al. 2021; Karakaş et al. 2022).

Our study found gender-based differences in the experience of stigma, with women reporting higher levels of stigma than men. This finding is consistent with some studies (Ayode et al. 2016), although other research has reported no significant gender differences (Boileau et al. 2020). In a study conducted in Africa, there were no significant gender differences in stigma perception, but it was observed that stigma experience differed between genders. Women were more likely to be concerned about physical appearance and life changes, while men were more concerned about sexual performance and economic prospects (Vlassoff et al. 2000). Although stigma experience heavily depends on culture (Karşidağ et al. 2019), this could be an explanatory hypothesis for the differences found in our sample. The questionnaire did not address topics such as sexual performance and economic difficulties but focused more on life changes and physical appearance alterations.

Additionally, we didn’t find differences in the perception of social and institutional support between genders. Other studies indicate that women report better quality of social support than men (Kneavel 2021) and both give and receive more social support (Tifferet 2020). One hypothesis to explain these results relates again to patient support associations. In our sample, we found no gender differences in the level of involvement with these associations, and as mentioned earlier, these associations provide information, advocacy, and support (Lobban and Camm 2011), which may contribute to a higher perception of social and institutional support.

We also observed a weak negative correlation between stigma experiences and the age of diagnosis, as well as the age of symptom onset. This pattern has also been identified in the literature, where longer disease duration is associated with greater stigma experience (Hou et al. 2021). Given that individuals with inherited diseases experience stigma, longer disease duration may suggest more instances of stigma throughout the patient's life and consequently higher scores in this subscale. Additionally, several studies in mental health suggest that the diagnosis itself increases experiences of stigma by enhancing social perceptions of group and individual differences (Corrigan 2007) This information leads us to hypothesize whether the same occurs in the case of inherited diseases. That is, if the diagnosis itself is a stigmatizing factor, prolonged experience with this label may contribute to the higher experiences of stigma found in this study.

The qualitative analysis of the participants’ accounts revealed the challenges participants face regarding the disclosure of information about the condition, and with obtaining a diagnosis. These findings align with prior research indicating that individuals with rare diseases often experience feelings of guilt regarding the heritability of their condition (Martinent et al. 2020), while families of affected individuals similarly report feelings of culpability for passing on the disease to their children (Marsh et al. 2011; Magliano et al. 2014; Manz 2016; Faure et al. 2019).

Participants reported stigma in various contexts, including healthcare settings, the workplace, and broader social interactions. These findings are consistent with literature showing that individuals with rare and hereditary diseases often experience structural stigma. For example, patients report feeling discredited by healthcare professionals and discriminated in social and professional settings (Wolfgang et al. 2023; Baynam et al. 2024). The stigmatizing experiences documented in our study, ranging from lack of empathy in healthcare settings to insufficient support from Social Security, may reflect a broader societal issue of misinformation and prejudice toward individuals with rare diseases. Similar patterns of institutional and social stigma have been reported in other countries, such as Brazil, where individuals with Sickle Cell Disease experience discrimination based on physical appearance and face institutional biases within the healthcare system (Silva et al. 2013).

Moreover, reports indicated significant emotional and psychological impacts, emphasizing the challenges in managing the illness and concerns about the future. The need for resilience in addressing these challenges has also been underscored. All of these findings are consistent with existing literature. Individuals from diverse backgrounds facing various hereditary diseases experience worry and uncertainty about their future (Higa et al. 2016), alongside feelings of sadness, anguish, indifference, guilt, shame and low self-esteem (Silva et al. 2013; Blake et al. 2018). They also encounter difficulties in managing chronic symptoms, adhering to treatment regimens, and coping with the uncertainties of disease progression (Silva et al. 2013). Nonetheless, instances of feeling resilient have been observed in response to negative encounters and experiences of stigma (Anderson et al. 2019). Parents of individuals with rare diseases frequently express frustration due to feelings of isolation and a lack of knowledge (Baynam et al. 2024). Fathers often experience anger and anxiety concerning their children's future, while mothers tend to prioritize their children's current well-being and feel a sense of responsibility for their condition (Cardinali et al. 2019). It is plausible that individuals with hereditary diseases often experience significant burdens, characterized by physical challenges exacerbated by social stigmatization, emotional and psychological distress, and a perceived lack of support. Collectively, these factors could diminish quality of life and hinder effective treatment adherence. Bogart and Irvin (2017) noted, living with a rare disease poses a great threat to Health-Related Quality of Life.

Interestingly, our study also highlighted the role of coping strategies, particularly the use of faith and humor. These findings echo previous research showing that parents of children with rare diseases often turn to religious coping mechanisms (Picci et al. 2015), and individuals with disabilities employ humor to navigate both personal and social stigma (Lash 2022). These strategies may serve as helpful tools for individuals in managing their psychological and emotional well-being, helping them to buffer the negative impacts of stigma and maintain a sense of control over their circumstances.

## Limitations and future research

The study has several limitations. The sample may not represent the broader population of individuals with hereditary diseases in Portugal, as those more severely affected may not have participated. Recruiting participants from patient associations could introduce bias, as these individuals may experience less stigma. Future research should compare stigma experiences across different diseases and include control groups not involved in patient associations to assess the protective effects of such involvement. Additionally, the diverse nature of hereditary diseases represented in our sample may lead to a highly heterogeneous depiction of the stigma experienced by participants. This variability may dilute disease-specific insights and complicate the generalization of findings. However, this heterogeneity can also be seen as a strength of the study, as it allows for the mapping of a broader spectrum of experiences, providing a more comprehensive understanding of the impact of stigma across a range of hereditary conditions. Finally, the small sample size limited the effectiveness of the EFA, and future studies should aim to validate the questionnaire for family members and asymptomatic carriers.

## Implications for practice

The results of this study highlight the need for comprehensive interventions to improve the well-being of individuals with hereditary diseases and their families. Systemic psychological interventions could help address the emotional and mental health needs of both affected individuals and their families. Family-centered interventions, such as multifamily discussion groups, may facilitate communication and support, helping families cope with the psychosocial challenges associated with hereditary diseases (Guerra et al. 2023).

Moreover, community engagement initiatives, facilitated through patient associations, should be encouraged to create peer support networks and advocate for increased resources and public awareness about hereditary diseases.

## Conclusion

This study is the first to report the experiences with stigmatization of individuals affected by hereditary diseases and family members in Portugal. It highlights the complex interplay between stigma, perceived support, and coping strategies, offering insights for interventions aimed at improving the quality of life of this group. By addressing both the psychosocial challenges and the need for enhanced support systems, we can contribute to fostering the well-being and resilience of individuals living with hereditary diseases.

## Supplementary Information

Below is the link to the electronic supplementary material.

Supplementary file1 (PDF 175 KB)

## Data Availability

No datasets were generated or analysed during the current study.

## Declarations

All procedures followed were in accordance with Portuguese legislation and the Helsinki Declaration of 2000. The study received ethics approval from i3S’s Committee for Ethical and Responsible Conduct of Research (N22 /CECRI/2023). Informed consent was obtained from all participants included in the study. No non-human animal studies were carried out by the authors for this study.
