# Supplementary material for: Experiences of stigmatization and its impacts among individuals living with hereditary diseases and family members in Portugal: an exploratory study
Source: J Community Genet. 2025 Feb 28;16(6):861–72. doi: 10.1007/s12687-025-00782-7 (PMC12569322; doi:10.1007/s12687-025-00782-7)
Supplement: Supplementary file 1 — Supplementary file1 (PDF 175 KB) [file 12687_2025_782_MOESM1_ESM.pdf]

Journal of Community Genetics

Title:

Experiences of Stigmatization and Its Impacts Among Individuals Living with Hereditary Diseases and Family Members in Portugal: An Exploratory Mixed-Method Study

Authors:

Joana Valentim [1] (https://orcid.org/0009-0005-6993-8720)
Milena Paneque [2,3,4] (https://orcid.org/0000-0002-6535-4315)
Álvaro Mendes [3,4] (https://orcid.org/0000-0002-8766-7646)

[1] Faculty of Psychology and Educational Sciences of University of Coimbra, Coimbra, Portugal
[2] CGPP – Centre for Predictive and Preventive Genetics, IBMC – Institute for Molecular and Cell Biology, University of Porto, Porto, Portugal
[3] i3S – Instituto de Investigação e Inovação em Saúde, University of Porto, Portugal
[4] ICBAS – Instituto de Ciências Biomédicas Abel Salazar, University of Porto, Porto, Portugal

Corresponding Author: Joana Valentim; Email: uc2022182215@student.uc.pt

Sociodemographic Questionnaire

1. Age category:
   - ☐ 18 – 23 years
   - ☐ 24 – 29 years
   - ☐ 30 – 35 years
   - ☐ 36 – 41 years
   - ☐ 42 – 47 years
   - ☐ 48 – 53 years
   - ☐ 54 – 59 years
   - ☐ 60 – 65 years
   - ☐ 66 – 71 years
   - ☐ 72 – 77 years

□ 78 – 83 years

□ 83 + years

2. Gender
   - Woman
   - Man
   - Other: ______
3. Marital Status
   - Single
   - Married/partnered
   - Divorced
   - Widowed
4. What is the highest level of education you have completed?
   - None
   - Primary education (up to 4 years of schooling)
   - Lower secondary education (5 to 6 years of schooling)
   - Upper secondary education (7 to 9 years of schooling)
   - Secondary education (10 to 12 years of schooling)
   - Bachelor's degree
   - Master's degree
   - Doctorate
   - Other. What? ____________
5. Area of residence
   - Rural
   - Urban
6. Employment status
   - Student
   - Unemployed
   - Employed
   - Retired
7. Do you have children??
   - Yes
   - No
8. How many?
   - _____
9. Do you intend to have children in the future?
   - Yes
   - No
   - I don’t know
10. Who is in your household (select all that apply):
    - Partner
    - Child(ren)
    - Father
    - Mother

- Sibling(s)
- Other:__________

11. [If you selected "Child(ren)"] How many children live with you?
    - _______
12. Through which support association for individuals and families living with hereditary diseases did you learn about this study?
13. What is your level of involvement with an organization supporting patients and families living with hereditary diseases?
    - None (even though I am associated, I have no contact with the association)
    - Rarely (I have contact with the association less than once a month)
    - Occasionally (I have contact with the association 2 to 3 times a month)
    - Frequently (I have contact with the association once a week)
    - Very frequently (I have contact with the association more than once a week)
14. What hereditary disease has been diagnosed in your family? _______________
15. I… (Select all that apply)
    - I am at risk of inheriting the genetic alteration that causes the disease
    - I have symptoms of the disease
    - I carry the genetic alteration that causes the disease, but I have no symptoms
    - I am a biological relative of someone with the disease or of a carrier of the genetic alteration that causes the disease
    - I am a non-biological relative of someone with the disease or of a carrier of the genetic alteration that causes the disease
16. At what age were you diagnosed with the genetic alteration? (If this question does not apply to your situation, click Next)
17. At what age did you first start experiencing symptoms? (If this question does not apply to your situation, click Next)
18. I am the primary caregiver for someone with a hereditary disease:
    - Yes
    - No

Questionnaire Group I – Individuals with a Clinical Diagnosis

1. [I am satisfied with my social support network.]
2. [Because of my illness, I feel that my family treats me differently.]
3. [I feel that knowing I have this illness would affect someone's desire to have an intimate relationship with me.]
4. [My hereditary illness did not/does not affect my desire to have children.]
5. [My family relationships have strengthened due to the way my family deals with the hereditary illness.]
6. [I feel I can openly talk about the hereditary illness with my family members.]
7. [Because of the hereditary illness, I had/have fewer job opportunities.]
8. [At work, people undervalue the challenges I face with my illness.]

9. [I face prejudice or discrimination at my workplace due to my illness.]
10. [I feel that I have the necessary support at work to deal with the challenges related to my illness.]
11. [I feel that my illness has helped me to choose my friendships more carefully.]
12. [I feel that knowing about my illness would affect someone's desire to be my friend.]
13. [My friends help me deal with the challenges related to my illness.]
14. [My illness has negatively affected my participation in social activities.]
15. [Society gives me the support necessary to cope with the challenges related to my hereditary illness.]
16. [I feel calm about my future.]
17. [I often feel sad because of my illness, even when I try to be optimistic.]
18. [I feel comfortable with others knowing about my illness.]
19. [I avoid making new friends so I don't have to explain my illness to them.]
20. [I have experienced negative reactions from others in social situations because of my illness.]
21. [When I feel someone treats me poorly because of my illness, I downplay what they say or do.]
22. [When I found out about my illness, I was worried about my family finding out.]
23. [I feel comfortable with others' attitudes toward my hereditary illness.]
24. [Some people avoid me because of my illness.]
25. [I feel comfortable sharing information about my hereditary illness with healthcare professionals.]
26. [Healthcare professionals understand the challenges I face in society due
27. to my hereditary illness.]
28. [Sometimes, I feel ashamed because of my illness.]
29. [My ability to face challenges related to my illness has improved over time.]
30. [I seek information that helps me cope with my illness.]
31. [I have sought psychological support to help me cope with my illness.]
32. [I have felt relieved by comparing my situation with others who have the same illness.]
33. [I have felt the need to hide information about my illness.]
34. [I feel that Social Security provides me with the necessary support to cope with my illness.]

Questionnaire Group II – Family Members

1. [I am satisfied with my social support network.]
2. [Due to my family's health condition, I feel there is a change in how I am treated.]
3. [I feel that having a hereditary illness in my family could affect someone's interest in establishing an intimate relationship with me.]
4. [The fact that someone in my family has the illness did not/does not affect my desire to have children.]

5. [My family relationships have strengthened due to how my family deals with the illness.]
6. [I feel I can openly discuss the illness with other family members.]
7. [Because of my family's hereditary illness, I believe I had fewer job opportunities.]
8. [At work, people undervalue the challenges I face due to my family's hereditary illness.]
9. [I face prejudice or discrimination at work due to my family's hereditary illness.]
10. [I feel I have the necessary support at my workplace to deal with the challenges related to my family's hereditary illness.]
11. [I feel that my family's hereditary illness has made me more selective about the friendships I maintain.]
12. [I feel that knowing about my family's hereditary illness would affect someone's desire to be my friend.]
13. [My friends help me deal with the challenges related to my family's hereditary illness.]
14. [My family's hereditary illness has negatively affected my participation in social activities.]
15. [Society provides me with the support necessary to cope with the challenges related to my family's hereditary illness.]
16. [I feel calm about my future.]
17. [I often feel sad because of my family's illness, even when I try to be optimistic.]
18. [I feel comfortable with others knowing about my family's hereditary illness.]
19. [I avoid making new friends so I don't have to explain my family's hereditary illness to them.]
20. [I have experienced negative reactions from others in social situations due to my family's hereditary illness.]
21. [When I feel that someone treats me poorly because of my family's hereditary illness, I downplay what they say or do.]
22. [When I found out about the illness in the family, I was worried about sharing the news with other family members.]
23. [I feel comfortable with others' attitudes toward my family's hereditary illness.]
24. [Some people avoid me because of my family's hereditary illness.]
25. [I feel comfortable sharing information about the hereditary illness with healthcare professionals.]
26. [Healthcare professionals understand the challenges my family faces in society due to the hereditary illness.]
27. [Sometimes, I feel ashamed because of my family's illness.]
28. [My ability to face the challenges related to the illness has improved over time.]
29. [I seek information that helps me deal with my family's hereditary illness.]
30. [I have sought psychological support to help me cope with my family's hereditary illness.]

31. [I have felt relieved by comparing my family's situation with other families who have the same illness.]
32. [I have felt the need to hide information about my family's hereditary illness.]
33. [I feel that Social Security provides me with the necessary support to cope with my family's hereditary illness.]

Questionnaire Group III – Asymptomatic Individuals

1. [I am satisfied with my social support network.]
2. [Because of my genetic mutation, I feel that I am treated differently within my family.]
3. [I feel that knowing I have this genetic mutation would affect someone's desire to have an intimate relationship with me.]
4. [My genetic mutation did not/does not affect my desire to have children.]
5. [My family relationships have strengthened due to how my family deals with the genetic mutation.]
6. [I feel I can openly talk about the genetic mutation with my family members.]
7. [Because of the genetic mutation, I had/have fewer job opportunities.]
8. [At work, people undervalue the challenges I face due to my genetic mutation.]
9. [I face prejudice or discrimination at my workplace due to my genetic mutation.]
10. [I feel that I have the necessary support at work to deal with the challenges related to my genetic mutation.]
11. [I feel that my genetic mutation has made me more selective about my friendships.]
12. [I feel that knowing about my genetic mutation would affect someone's desire to be my friend.]
13. [My friends help me deal with the challenges related to my genetic mutation.]
14. [My genetic mutation has negatively affected my participation in social activities.]
15. [Society gives me the support necessary to cope with the challenges related to my genetic mutation.]
16. [I feel calm about my future.]
17. [I often feel sad because of my genetic mutation, even when I try to be optimistic.]
18. [I feel comfortable with others knowing about my genetic mutation.]
19. [I avoid making new friends so I don't have to explain my genetic mutation to them.]
20. [I have experienced negative reactions from others in social situations due to my genetic mutation.]
21. [When I feel that someone treats me poorly because of my genetic mutation, I downplay what they say or do.]
22. [When I found out about my genetic mutation, I was worried about sharing the news with my family.]
23. [I feel comfortable with others' attitudes toward my hereditary genetic mutation.]
24. [Some people avoid me because of my genetic mutation.]

25. [I feel comfortable sharing information about my genetic mutation with healthcare professionals.]
26. [Healthcare professionals understand the challenges I face in society due to my genetic mutation.]
27. [Sometimes, I feel ashamed because of my genetic mutation.]
28. [My ability to face the challenges related to my genetic mutation has improved over time.]
29. [I seek information that helps me cope with my genetic mutation.]
30. [I have sought psychological support to help me cope with my genetic mutation.]
31. [I have felt relieved by comparing my situation with others who have the same genetic mutation.]
32. [I have felt the need to hide information about my genetic mutation.]
33. [I feel that Social Security provides me with the necessary support to cope with my genetic mutation.]

Open-ended question

Each questionnaire concluded with the following open-ended question: If you wish, use the space below to share any specific experience or additional detail you consider relevant or that was not addressed in the previous questions. If you prefer not to provide additional comments, click Submit.
